# Endothelin Blockade in Diabetic Kidney Disease

**DOI:** 10.3390/jcm4061171

**Published:** 2015-05-25

**Authors:** Lidia Anguiano, Marta Riera, Julio Pascual, María José Soler

**Affiliations:** 1 Department of Nephrology, Hospital del Mar-IMIM (Hospital del Mar Medical Research Institute), 88 Dr. Aiguader Street, Barcelona, 08003, Spain; E-Mails: languiano@imim.es (L.A.); mriera1@imim.es (M.R.); JPascualSantos@parcdesalutmar.cat (J.P.); 2 Red de Investigación Renal (REDINREN), Instituto Carlos III-FEDER, Madrid 28029, Spain

**Keywords:** diabetic kidney disease (DKD), endothelin-1 (ET-1), endothelin A receptor (ET_A_ receptor), endothelin B receptor (ET_B_ receptor), endothelin receptor antagonists

## Abstract

Diabetic kidney disease (DKD) remains the most common cause of chronic kidney disease and multiple therapeutic agents, primarily targeted at the renin-angiotensin system, have been assessed. Their only partial effectiveness in slowing down progression to end-stage renal disease, points out an evident need for additional effective therapies. In the context of diabetes, endothelin-1 (ET-1) has been implicated in vasoconstriction, renal injury, mesangial proliferation, glomerulosclerosis, fibrosis and inflammation, largely through activation of its endothelin A (ET_A_) receptor. Therefore, endothelin receptor antagonists have been proposed as potential drug targets. In experimental models of DKD, endothelin receptor antagonists have been described to improve renal injury and fibrosis, whereas clinical trials in DKD patients have shown an antiproteinuric effect. Currently, its renoprotective effect in a long-time clinical trial is being tested. This review focuses on the localization of endothelin receptors (ET_A_ and ET_B_) within the kidney, as well as the ET-1 functions through them. In addition, we summarize the therapeutic benefit of endothelin receptor antagonists in experimental and human studies and the adverse effects that have been described.

## 1. Introduction

Diabetic kidney disease (DKD) is the most common cause of chronic kidney disease, leading to premature death and end-stage renal disease in the developed and developing world. Therefore, multiple potential therapeutic agents have been studied, focusing on the treatment of hyperglycemia and hypertension mainly focused on the renin-angiotensin system blockade [1]. However, these therapies only partially slow down progression to end-stage renal disease, thus there is a need for additional effective therapies. In this context, the blockade of the endothelin (ET) system has emerged as new potential strategy.

ET-1 was first reported by Hickey *et al.* [2] in 1985 as an endothelial cell-derived peptide. The ET system is a family of 21 amino acid peptides, comprising ET-1, ET-2 and ET-3 [3], with powerful vasoconstrictor and pressor properties. ET-1 and ET-2 differ in two nonpolar amino acids, while ET-3 isoform differs in more amino acids compared to the two other isoforms. ET-1 is the predominant endothelin isoform present in the human kidney [4,5], produced by mesangial and glomerular epithelial cells and renal tubular and medullary collecting duct cells [6].

ET-1 acts via two G-protein-coupled receptors, ET_A_ and ET_B_, which are highly expressed in the kidney. ET receptors are widely distributed within the human kidney. The ET_A_ receptor was localized in vascular smooth muscle, in the glomeruli, vasa recta and arcuate arteries, adjacent veins and arterioles. The ET_B_ receptor is heterogeneously distributed with high expression in glomerular endothelial cells as well as epithelial cells lining the renal tubule, particularly in the collecting ducts [7]. ET receptors seem to have quite opposite functions. ET_A_ receptor activation results in increased oxidative stress, over-expression of circulating and glomerular inflammatory mediators as well as changes in glomerular permeability to albumin [8,9,10]. In contrast, ET-1 via ET_B_ results in vasodilatory, antiproliferative and antifibrotic effects [11]. It has been previously shown that under pathological conditions associated with renal disease, such as diabetes and hypertension, renal ET-1 production increases [12]. This increase induces to vasoconstriction, podocyte injury, mesangial proliferation, matrix accumulation, glomerulosclerosis, fibrosis and inflammation through the ET_A_ receptor [10].

Taken together, ET-1 has a crucial role in the development of kidney disease through the ET_A_ receptor becoming an attractive therapeutic target in various forms of renal diseases, such as DKD. Therefore, ET receptor antagonists have been largely proposed and studied for the treatment of renal diseases. Several experimental studies and some clinical trials have shown that ET receptor antagonists ameliorate DKD, but adverse effects, such as fluid retention have been also described.

In this review we will describe the ET receptors localization within the kidney. In addition, we will focus on the endothelin receptor antagonists that have been or are being studied for the treatment of DKD and its adverse effects.

## 2. Endothelin Receptors in the Kidney

**Figure 1:**
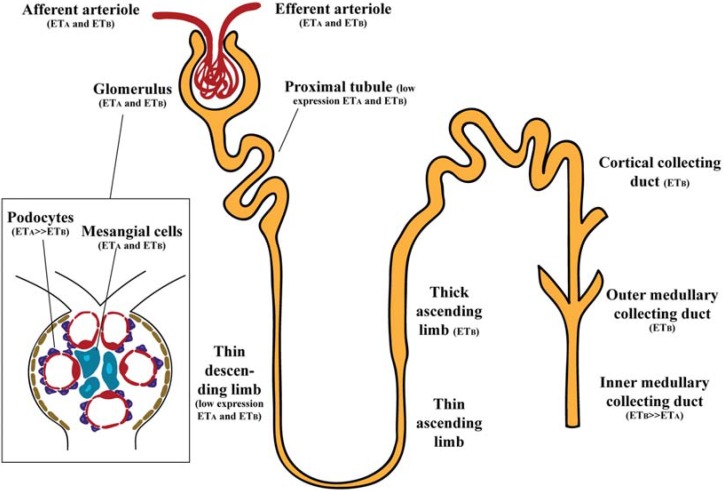
Schematic representation of functional ET-1 receptors in the kidney. Glomerulus (podocytes and mesangial cells) express primarily ET_A_ receptors. In renal microcirculation both ET_A_ and ET_B_ receptors are expressed. Renal tubules contain mainly ET_B_ receptors, with more expression in the thick ascending limb and the collecting duct.

ET receptors are widespread within the kidney, and it has been described to be 10 times more sensitive to the vascular effects of ET-1 than in other organs [13]. ET_A_ and ET_B_ receptors do not have the same expression in all regions of the kidney (Figure 1). Studies conducted in human kidney suggested that renal cortex and medulla contain ET_A_ and ET_B_ receptors in a ratio of 30:70 and that ET-1 binds to both receptors with the same high affinity [14].

### 2.1. Glomerulus

The ET system is present throughout all the glomerulus. Quantitative analysis of ET binding sites in rat kidney suggested abundance of ET-1 in glomeruli, with more ET-1 expression present in podocytes than in mesangial cells [15]. In human kidney grafts, ET-1, ET_A_ and ET_B_ receptors were found in the glomeruli [16]. ET_A_ receptors seem to be more expressed in podocytes, since effects of ET-1 were prevented by ET_A_, but not ET_B_ antagonists [17], however immunoelectron microscopy localized ET_B_ in rat podocytes [18]. In mesangial cells, both ET_A_ and ET_B_ receptors have been detected by immunofluorescence in rat kidney [19]. In concordance, *in vitro* studies also confirmed the presence of ET_A_ and ET_B_ receptors in human mesangial cells [20,21]. 

### 2.2. Renal Vasculature

In the renal vasculature, ET_A_ receptors are mainly localized on vascular smooth muscle of afferent and efferent arterioles (but not on endothelial cells) and in pericytes of the descending vasa recta bundles, whereas ET_B_ receptors are predominantly located on endothelial cells of afferent and efferent arterioles and vasa recta [19]. 

### 2.3. Renal Tubules

Although proximal tubule (PT) expresses ET receptors, there is low affinity of ET-1 binding in PT segments [22,23]. The PT expresses both ET_A_ and ET_B_ receptors; however their expression seems to be low. Initially, Terada *et al.*, were unable to detect ET_B_ receptor mRNA in rat PT segment [24], but subsequent studies were able to identify ET_B_ immunoreactivity [19,25]. In addition, Yamamoto *et al.* reported ET_A_ receptor immunoreactivity in rat PT cells [26].

Little is known about the biology of ET in the thin limb of Henle’s loop. ET-1 expression was detected in the thin descending limb of rat kidney, but at lower levels than any other segment of the nephron [27]. Few studies have been performed addressing ET receptors in the thin descending limb, but no data has been reported about thin ascending limb. Only one study was able to detect the ET_A_ and ET_B_ receptor mRNA expression in the rat thin descending limb of long-looped nephrons [28]. Later studies were unable to detect immunoreactivity to ET receptors in this segment of the nephron [19,26]. Some studies failed to observe evidence for ET receptors in the thick ascending limb [19,29]. However, others found only ET_B_ expression [24,30].

ET-1 was found in human inner medullary collecting duct (IMCD) cells [31] and human and pig kidneys were shown to synthesize and release ET-1 [32]. Within the kidney it seems that ET receptor expression is mainly located in the CD. Specifically, the highest ET-1 binding has been found in the IMCD, while the outer medullary collecting duct (OMCD) and the cortical collecting duct (CCD) exhibit moderate binding [22]. Several studies have confirmed ET_B_ as the predominant receptor. Takemoto *et al.* demonstrated that ET_B_ antagonism inhibited ET-1 binding to the CCD, suggesting ET_B_ receptor as the major receptor of this segment of the nephron [22]. Porcine renal papillary membranes showed high affinity for ET_B_, but not for ET_A_, receptors [33]. The ET_B_ predominated over ET_A_ receptors (2:1) in the collecting system [7,34]. Relative quantification of the ET_B_ receptor mRNA in renal nephron segments showed higher expression in IMCD than ET_A_ receptor mRNA [24]. Immunofluorescence studies from rat kidneys revealed ET_B_ expression in the IMCD [19]. This study also found specific immunofluorescence for ET_A_ in the CCD [19].

## 3. Functions of Endothelin in the Kidney

**Figure 2:**
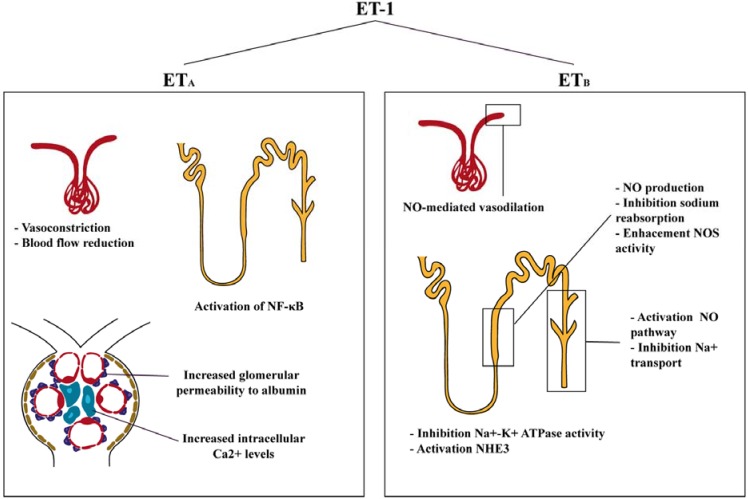
Effects of ET-1 on the kidney. ET-1 through ET_A_ receptors has vasoconstrictor, pro-inflammatory and podocyte-injury related effects. ET-1 activation through ET_B_ receptors leads to vasodilation and activation of NO pathway.

ET-1 actions within the kidney differ whether its effects are through ET_A_ or ET_B_ receptors. Binding to ET_A_ promotes vasoconstriction, cell proliferation, fibrosis and podocyte damage; while binding to ET_B_ promotes vasodilator, antiproliferative and antifibrotic effects. Interestingly, the ET_B_ receptor is mainly involved in regulation of fluid transport (Figure 2). Podocytes are an essential part of the glomerular filtration barrier and there is evidence that ET-1 may promote podocyte injury, aggravating albumin urinary loss and alteration of the glomerular microvasculature [35]. Morigi *et al.*, demonstrated that cells exposed to ET-1 lead to a F-actin redistribution at the cell periphery, providing the first evidence that ET-1 may alter podocytes F-actin contractile apparatus relevant for the maintenance of glomerular permselectivity [36]. Later studies in isolated glomeruli from hyperglycemic rats incubated with ET receptor antagonists, showed that the ET_A_ antagonist significantly reduced the elevated glomerular permeability to albumin, but the ET_B_ antagonist had no effect on the increased glomerular permeability to albumin in glomeruli [37]. In mesangial cells, ET-1 activates a wide variety of signaling pathways with alterations in cell contraction, hypertrophy, proliferation and extracellular matrix accumulation [38]. Mesangial cells incubation with ET-1 leads to a rapid rise in intracellular calcium [39] but expression of dihydropyridine-sensitive Ca^2+^ channels were not modified, suggesting that mesangial cell contraction is independent of these channels. ET-1 seemed to stimulate mesangial cell contraction via pharmacomechanical coupling and activates phospholipase A2 to produce PGE2, PGF2 alpha, and TXB2. ET-1 also amplified beta adrenergic-stimulated cAMP accumulation by a PGE2-dependent mechanism [40]. Yokokawa *et al.* showed that ET-1 increased intracellular Ca^2+^ level via the ET_A_ receptor, because an ET_A_ antagonist suppressed intracellular Ca^2+^ elevation in response to ET-1 [41]. Staining with rhodamine-phalloidin revealed complex ET-1 and Ca2+-mediated rearrangements of mesangial F-actin microfilament bundles [40].

In the renal microcirculation, a study using the blood-perfused juxtamedullary nephron preparation, that allows to identify site-specific actions of endothelin receptor activation, demonstrated that the afferent arteriole is more sensitive to the vasoconstrictor actions of ET-1 and that ET_A_ receptors mediate mainly vasoconstrictor actions in both the afferent and efferent arterioles [42]. Endogenous ET-1 acts through ET_A_ receptors to reduce medullary and cortical perfusion, thus reducing renal blood flow. However, no effects on the glomerular filtration rate (GFR) have been seen in any of these studies [43,44,45,46]. In contrast, the ET_B_ receptor mediated vasodilation in the efferent arteriole [42]. Schildroth *et al.* confirmed that ET_B_ receptor activation induces nitric oxide (NO)-mediated vasodilation in efferent arterioles [47]. In other experimental studies, Qiu *et al.* demonstrated that endogenous ET-1 contributed to the control of renal hemodynamics, and subsequently regulating glomerular pressure [48]. 

In renal tubules, ET-1 acts predominantly through the ET_B_ receptor pathway, resulting in inhibition of Na^+^-K^+^ ATPase activity, an effect blocked by ET_B_ antagonism [49,50]. In addition, ET_A_ activates the apical membrane Na/H antiporter (NHE3), relevant in the context of acidosis within the kidney [51,52]. In the medullary thick ascending limb, ET-1 activates phosphatidylinositol 3-kinase (PI3K), inducing phosphorylation and activation of Akt, which in turn phosphorylates eNOS, resulting in increased NO production and inhibition of sodium reabsorption [53]. This mechanism likely plays an important role in the regulation of thick ascending limb NO production and sodium homeostasis. In the cortical thick ascending limb, ET-1 inhibits transport by enhancing endogenous eNOS activity and releasing NO via activation of the ET_B_ receptors, an effect associated with increases in intracellular calcium [54,55]. Few studies have described a role for ET_A_ receptors in renal tubules. In particular, in human renal proximal tubular cells, activation of NF-κB, a signaling pathway implicated in progressive renal interstitial fibrosis, occurs through ET_A_ receptors [56].

A first study analyzing ET-1 actions in the CD found that, in suspensions of rabbit IMCD, ET-1 selectively reduced Na^+^-K^+^ ATPase activity [57]. Furthermore, they confirmed that the inhibition was independent of ATP synthesis, and ascribed to direct interaction with the pump. Later studies in isolated rat CD demonstrated that ET-1 exerts an inhibition of vasopressin, which is associated with an increase in sodium and chloride absorption in the CCD [58,59]. Since ET-1 is implicated in mediating CD sodium transport, further experimental studies where performed disrupting ET-1, ET_B_ or ET_A_ receptors specifically within principal cells of the CD. In ET-1 gene deleted mice, no differences were observed in urine sodium or potassium excretion when animals were fed with a normal diet. However, when a high-salt diet was administered, these mice showed reduced ability to excrete a sodium load associated with weight gain and worsened hypertension [60]. When the amiloride diuretic was administered blood pressure was reduced and excessive sodium retention was prevented in CD ET-1 KO mice, suggesting that ET-1 may regulate CD sodium absorption through modulation of sodium channels (ENaC) activity. In isolated principal cells of the rat CD, ET-1 decreased ENaC activity, probably through ET_B_ receptor [61]. Schneider *et al.* determined that urinary excretion of sodium was blunted in CD ET-1 KO mice and that the pressure-dependent changes in sodium and water excretion require ET-1 activation of the NO pathway (most likely nNOS and eNOS) [62]. *In vitro* studies have also shown that ET-1 stimulates NO in isolated IMCD cells through ET_B_ and not ET_A_ receptors. The effect of NO stimulation by ET-1 was abolished by the incubation with ET_B_ receptor antagonist, but not affected by an ET_A_ receptor antagonist [63]. In a study by Ge *et al.*, mice with deficiency of the ET_B_ receptor in the CD showed hypertension on a normal diet and elicited salt-sensitive hypertension associated with impaired ability to excrete a sodium load [64]. Cell-specific disruption of the ET_A_ gene (CD ET_A_ KO) was also analyzed and no effects in blood pressure and sodium excretion in mice on normal or high-salt diet were observed. These results demonstrated that the deleterious effects of ET-1 are mediated through the ET_A_ pathway.

## 4. Endothelin Antagonists in Diabetic Nephropathy

ET-1 has an important role in renal pathophysiology and a direct role in kidney disease has been reported. Numerous factors, such as hyperglycemia and hypertension [65], contribute to increased renal ET-1 production, and therefore progression of kidney disease, in diabetic nephropathy.

Renal overexpression of ET-1 induced an age-dependent development of renal cysts, glomerulosclerosis and interstitial fibrosis without hypertension. Furthermore, this pronounced renal fibrosis resulted in a significantly age-dependent decreased GFR leading to fatal kidney disease [66]. ET-1 contributes also to important hemodynamic effects that reduce renal blood flow and glomerular filtration at concentrations that do not alter blood pressure [67]. ET-1 induces the formation of angiotensin II, a vasoconstrictor peptide [68]. In addition, angiotensin II activates renal ET-1 formation [69], creating a positive feedback loop. A direct action of ET-1 on podocytes has been recently reported, suggesting that ET-1 drives the development of glomerulosclerosis and podocyte loss through direct activation of endothelin receptors [70]. Both hyperglycemia and ET-1 cause disassembly of the podocyte actin cytoskeleton, apoptosis and podocyte depletion [71].

Since activation of the ET-1 system plays an important role in renal kidney disease, and particularly in DKD, ET receptor antagonists have become potential therapeutic agents. Several ET receptor antagonists (ET_A_ receptor, ET_B_ receptor and ET_A_-ET_B_ receptor antagonists) have been developed: BQ-123, BQ-778, BMS182874, BQ-928, BQ-238, TAK044 and the novel “-sentan” class of drugs (bosentan, avosentan, darusentan, sitaxsentan and atrasentan). However, not all of them have been studied in the context of DKD, but in other types of kidney disease, mainly hypertensive chronic kidney disease (CKD) and non-diabetic CKD. The experimental (Table 1) and human (Table 2) studies involving diabetic nephropathy and ET receptor antagonists are summarized below.

### 4.1. Experimental Studies 

#### 4.1.1. BQ-123

BQ-123 was characterized as an ET_A_-selective ligand that competes for the binding of ET-1 both in medulla and cortex [34]. In the study by Granstam *et al.*, diabetic rats exhibited decrease renal blood flow as compared with non-diabetic rats, providing evidence of vasoconstriction in the context of diabetes. Treatment of diabetic rats with the ET_A_ receptor antagonist BQ-123 implied no alteration in renal blood flow [72]. However, in isolated glomeruli from diabetic rats albumin permeability was increased, while treatment with BQ-123 significantly reduced the albumin permeability in a dose-dependent manner [37]. The use of isolated glomeruli allows the measurement of glomerular capillary independent of changes in renal hemodynamics. This includes evidence of a direct, ET_A_ dependent increase in glomerular permeability and nephrin loss that occurs in the hyperglycemic kidney. In addition, ET_A_ receptor blockade provides anti-inflammatory actions by reducing hyperglycemia dependent increases in early inflammatory markers such as MCP-1 and sICAM-1. This study suggests that the protective effects of ET receptor antagonists on renal injury appear to be independent of blood pressure [37]. Studies in rat tubular epithelial cells treated with a high-glucose medium showed increased ET-1 levels and decreased levels of E-cadherin and vimentin expression, epithelial and mesenchymal markers. Treatment with BQ-123 prevented the changes in E-cadherin and vimentin, suggesting that ET-1 induces epithelial-mesenchymal transition through ET_A_ in renal tubular cells [73]. 

#### 4.1.2. BQ-788

The selective ET_B_ receptor antagonist BQ-788 has been studied together with the ET_A_ receptor antagonist BQ-123 in a study performed by Saleh *et al.* [37]. When isolated glomeruli of diabetic rats were treated with BQ-788 no effect on the elevated albumin permeability in the context of diabetes was observed. The combination of BQ-123 and BQ-788 reduced albumin permeability in a fashion similar to BQ-123 alone. In addition, studies in cultured podocytes demonstrated that exogenous ET-1 added to these cells, increased albumin permeability directly, an effect blocked by an ET_A_ but not ET_B_ antagonist. These results suggest that the effect of ET_B_ receptors to modulate proteinuria is via hemodynamic changes rather than direct effects on the filtration barrier [37].

#### 4.1.3. Bosentan

Bosentan was obtained by structural optimization of a less potent dual receptor antagonist (Ro 46-2005). It has been described as a non-peptide mixed antagonist of ET-1 with capacity of inhibiting the stimulation of both ET_A_ and ET_B_ receptors [74]. Experimental studies analyzing the effects of bosentan on DKD are controversial. Kelly *et al.*, showed that diabetic (mRen-2)27 rats exhibited increased albuminuria that was reduced by an AT1 receptor antagonist (valsartan). By contrast, bosentan, although normalizing blood pressure and improving GFR, had little or no effect on the renal lesions and was associated with increased albuminuria [75]. Interestingly, other studies, demonstrated that the administration of bosentan prevented the increase in urinary protein excretion in diabetic rats [76,77]. Combination of bosentan with a calcium channel blocker also attenuated the enhanced urinary protein excretion [78]. These contradictory results may be ascribed to the different animal models used for the studies. Kelly *et al.* studied hypertensive rats (with streptozotocin (STZ)-diabetes), while the other studies were performed in STZ rats and uninephrectomized-STZ rats. One might surmise, that the deleterious effect observed in the mRen-2 STZ model may be ascribed to the higher activation of the renin-angiotensin system.

Diabetes-induced fibrosis (increased fibronectin, TGFβ, collagen I, collagen IV and AP-1 transcription factor) was reduced by the administration of the dual receptor antagonist [76,78,79]. Cai *et al.* demonstrated that diabetes increased metallothionein (MT), a stress-responsive protein, which can be activated by cytokines such as TNFα. In this study, bosentan had a tendency to reduce MT. When comparing the effect of bosentan on renal structural injury in diabetic nephropathy, different results have been found. Ding *et al.* confirmed that bosentan prevented renal structural injury, while Kelly *et al.* concluded that bosentan not only did not attenuate structural injury, but displayed severe glomerulosclerosis and tubulointerstitial disease [75]. The incongruences found in the presented studies may be related to the blockade of both ET_A_ and ET_B_ receptors.

**Table 1:** ET-1 receptor antagonists in experimental diabetic nephropathy.

Drug	ET_A_/ET_B_ Affinity	Source	Type of Study	Experimental Model	Type of Diabetes	Main Outcomes
**BQ-123**	ET_A_	Simonson *et al.*, 1990 [40]	*In vitro*	Rat mesangial cells	-	Reduction of albumin permeability
		Granstam *et al.*, 2011 [72]	*In vivo*	STZ-induced diabetic Sprague Dawley rats	Type 1	No alteration on renal blood flow
		Tang *et al.*, 2014 [73]	*In vitro*	Rat tubular epithelial cells	-	Prevention of changes in E-cadherin and vimentin (epithelial-mesenchymal transition)
**BQ-778**	ET_B_	Saleh *et al.*, 2011a [37]	*In vitro*	STZ-induced diabetic Sprague Dawley rats	Type 1	No effect on elevated albumin permeability
						Reduction of albumin permeability in combination with BQ-123
**Bosentan**	ET_A_/ET_B_	Kelly *et al.*, 2000 [75]	*In vivo*	STZ-induced diabetic Ren-2 rats	Type 1	Increased albuminuria
						Attenuation of decrease in GFR
						Severe glomerulosclerosis and tubulointerstitial damage
		Tikkanen *et al.*, 2002 [104]	*In vivo*	STZ-induced diabetic Sprague Dawley rats	Type 1	No reduction in albuminuria
		Chen *et al.*, 2002 [78]	*In vivo*	STZ-induced diabetic hypertensive rats	Type 1	Prevention of urinary protein excretion
		Cosenzi *et al.*, 2003 [76]		STZ-induced diabetic Wistar Kyoto rats		Reduction in diabetes-induced fibrosis
		Ding *et al.*, 2003 [77]		Uninephrectomized STZ-induced diabetic rats		Prevention of renal injury
**Darusentan**	ET_A_	Hocher *et al.*, 1998 [80]	*In vivo*	STZ-induced diabetic rats	Type 1	Reduction in urinary protein excretion
		Dhein *et al.*, 2000 [81]		STZ-induced diabetic Wistar Kyoto rats		Prevention of glomerulosclerosis index, tubulointerstitial damage index and glomerular volume
		Gross *et al.*, 2004a/b [82,83]		SHR/N-corpulent rats		Ineffective in prevention of podocyte loss and damage
**Avosentan**	ET_A_	Gagliardini *et al.*, 2009 [84]	*In vivo*	STZ-induced diabetic Sprague Dawley rats	Type 1	Reduction in urinary protein excretion
						Reduction of glomerulosclerosis, tubulointerstitial damage and mesangial expansion
						Reduction of accumulation of inflammatory cells and staining of TGFβ and collagen deposition
						No reduction of glomerular hypertrophy Increase in nephrin protein expression
		Watson *et al.*, 2010 [85]	*In vivo*	STZ-induced diabetic *ApoE* KO mice	Type 1	Reduction in urinary protein excretion
						Reduction on gene expression levels of fibronectin, collagen IV, TGFβ and α.SMA
**Sitaxentan**	ET_A_	Zoja *et al.*, 2011 [86]	*In vivo*	Zucker Diabetic Fatty rats	Type 2	No effect on albuminuria and glomerulosclerosis
						Decrease in systolic blood pressure Reduction in protein matrix accumulation
**Atrasentan**	ET_A_	Sasser *et al.*, 2007 [89]	*In vivo*	STZ-induced diabetic Sprague Dawley rats	Type 1	Attenuation of urinary excretion of TGFβ
						No effects in reactive oxygen species production
		Saleh *et al.*, 2011a [37]	*In vivo/In vitro*	STZ-induced diabetic Sprague Dawley rats/Isolated glomeruli	Type 1	Reduction in proteinuria and albumin permeability
		Saleh *et al.*, 2011b [88]	*In vivo/In vitro*	STZ-induced diabetic Sprague Dawley rats/Isolated glomeruli	Type 1	Reduction in proteinuria and albumin permeability
						Prevention of proinflammatory molecules increase
						Increase in gene expression levels of nephrin, ZO-1 and podocin

**Table 2:** ET-1 receptor antagonists in human studies and clinical trials.

Drug	ET_A_/ET_B_ Affinity	Source	Type of Study	Subjects (Completed Study)	Type of Diabetes	Dosage	Main Outcomes	Adverse Effects
**Bosentan**	ET_A_/ET_B_	Rafnsson *et al.*, 2012 [92]	Randomized, double-blind, placebo-control trial	46	Type 2	62.5 mg daily-2 weeks + 125 mg twice daily-2 weeks (in absence of side effects)	No changes in urine ACR ratio, blood pressure and blood glucose Increase in ET-1 plasma levels	One patient with edema (discontinued intervention)
**Avosentan**	ET_A_	Wenzel *et al.*, 2009 [93]	Randomized, double-blind, placebo-controlled, dosage-range, parallel-group phase 2 study	252	Type 1 and 2	5, 10, 25 and 50 mg (12 weeks)	Decrease in urinary albumin excretion rate (−20.9% to −29.9%) Reduction in urinary protein excretion	Dosage-dependent fluid retention (32.1% of patients in 50 mg dosage)
		Mann *et al.*, 2010 [94]	International, multicenter, randomized, double-blind phase 3 clinical trial	1392	Type 2	25 and 50 mg (prematurely terminated)	ACR declined in a range of 40%–50% in avosentan groups No changes in blood pressure	Increased early mortality mainly due to fluid overload and congestive heart failure. Prematurely terminated.
**Atrasentan**	ET_A_	Kohan *et al.*, 2011 [95]	Randomized, double-blind, placebo-controlled phase 2a clinical trial	81	Type 2	0.25, 0.75 and 1.75 mg (8 weeks)	Up to 42% ACR reduction in atrasentan groups	Dose-dependent peripheral edema One patient with serious adverse effect (elevated baseline NT-pro BNP)
		Andress *et al.*, 2012 [96]	Randomized, double-blind, placebo-controlled phase 2a clinical trial	89	Type 2	0.25, 0.75 and 1.75 mg (8 weeks)	Up to 40% ACR reduction in atrasentan groups	Associated with 1.75 mg treatment group and baseline urinary ACR
		de Zeeuw *et al.*, 2014 [97]	Data pooled from two phase2b studies	183	Type 2	0.75 and 1.25 mg/day (12 weeks)	Up to 39% ACR reduction in atrasentan groups	Higher number of patients discontinued due to fluid retention-related events in 1.75 mg *vs*. 0.75 mg group
		SONAR (actively enrolling)	Phase 3 clinical trial	4148 (estimated enrolling)	Type 2	Low dose (48 months)	Ongoing	Ongoing

#### 4.1.4. Darusentan

Darusentan has been described as an ET_A_ receptor antagonist. Studies in type 1 and type 2 diabetic animals showed that darusentan had a tendency to reduce urinary protein excretion [80,81,82]. Increased glomerulosclerosis index, tubulointerstitial damage index and glomerular volume were observed in STZ-untreated rats. All these effects were reduced by treatment with ET_A_ receptor antagonist [81,82,83]. However, studies by Gross *et al.* in type 1 and type 2 diabetic rats demonstrated that the loss of podocytes, podocyte damage and abnormal podocyte phenotype could be prevented by treatment with an angiotensin converting enzyme inhibitor (ACEi), but administration of darusentan was ineffective [82,83].

#### 4.1.5. Avosentan

Avosentan is an ET_A_ receptor antagonist with ~500-fold selectivity for ET_A_ over ET_B_ receptor. This ET_A_ receptor antagonist has been studied alone and in combination with ACEi (lisinopril or quinapril) in STZ-diabetic rats and STZ-ApoE KO mice [84,85]. In both studies, high blood pressure was not decreased by treatment with avosentan, while ACEi treatment significantly reduced blood pressure. Urinary protein excretion was reduced by administration of avosentan, although in diabetic rats only a tendency was observed. Regarding renal injury, avosentan reduced glomerulosclerosis, tubulointerstitial damage and mesangial expansion as compared to untreated diabetic animals. Furthermore, avosentan decreased interstitial accumulation of inflammatory cells and staining of TGFβ, collagen deposition [84], gene expression levels of fibronectin, collagen IV, TGFβ, and α-SMA [85]. Gagliardini *et al.* also studied the integrity of podocytes in STZ rats. Interestingly, avosentan alone or in combination with ACEi restored the number of podocytes per glomerulus, as well as nephrin protein expression. This suggests that the effect of avosentan of limiting podocyte loss may be associated with the normalization of nephrin levels [84]. Taken together it seems that avosentan and mainly the combination with an ACEi, diminished proteinuria and provided renal protection from glomerular and tubulointerstitial injury in DKD.

#### 4.1.6. Sitaxsentan

There is only one study associating sitaxsentan (an ET_A_ receptor antagonist) with the Zucker rat model of type 2 diabetes [86]. In this study, sitaxsentan failed to reduce albuminuria and glomerulosclerosis, but had a significant effect on lowering systolic blood pressure. In addition, sitaxsentan alone reduced protein matrix accumulation, represented by type III collagen depositions and renal inflammation (MCP-1 gene expression and number of macrophages). Combination of sitaxsentan with an ACEi afforded renoprotection in this type 2 diabetes animal model, but the effect was mainly due to the ACEi [86].

#### 4.1.7. Atrasentan

Atrasentan is a highly selective inhibitor of ET_A_ receptors (1800-fold ET_A_ > ET_B_) that decreases the binding affinity of ET-1 without affecting receptor density and thus competitively blocks the effects of ET-1 receptor binding [87]. In STZ-diabetic rats, atrasentan produced a significant decrease in proteinuria observed earlier, after only one day of treatment, while the ET_B_ receptor antagonist, A-182086, had no effect [88]. After three or six weeks of treatment, proteinuria was also decreased [37]. Saleh *et al.* demonstrated that atrasentan decreases albumin permeability after one, three or six weeks of treatment. Interestingly, albumin permeability was highly correlated to proteinuria [37,88]. Atrasentan also exerted an anti-inflammatory effect, demonstrated by the prevention of the increase in circulating ICAM-1 and MCP-1 observed in this diabetic model [37,88]. In addition, atrasentan attenuated the increase of urinary and glomerular TGFβ [88,89]. Within the podocyte, Saleh *et al.* demonstrated that gene/protein expression of nephrin, ZO-1 and podocin [37,88] were restored to control levels in diabetic atrasentan-treated rats. Diabetes-induced increase in urinary excretion of nephrin was also prevented by the ET_A_ receptor antagonist. 

### 4.2. Human Studies and Clinical Trials

#### 4.2.1. Bosentan

The dual receptor antagonist, bosentan, is a compound approved for the treatment of pulmonary arterial hypertension and digital ulcers in scleroderma [90,91]. In DKD, only one clinical trial was performed using this type of antagonist. The BANDY (Effect of Bosentan on Endothelial Function in Patients With Type 2 Diabetes) was a randomized, double-blind and placebo-control trial, that includes patients with type 2 diabetes of at least two years of duration and microalbuminuria [92]. These patients received bosentan in a dosage of 62.5 mg daily for two weeks and, in the absence of side effects, the dosage was titrated to 125 mg twice daily for two weeks. Treatment with bosentan showed no significant changes in urine albumin-to-creatinine ratio (ACR), blood pressure and blood glucose. A significant increase in plasma ET-1 levels and a significant drop in hemoglobin in patients in bosentan group were observed. There was only one adverse effect related with edema and the patient had to discontinue the intervention. 

#### 4.2.2. Avosentan

One phase 2 and phase 3 studies have been performed involving the ET_A_ receptor antagonist, avosentan, and patients with diabetic nephropathy. The first study was a randomized, double-blind, placebo-controlled, dosage-range, parallel-group phase 2 study, where type 1 and 2 diabetic patients were treated with different dosages of avosentan (5, 10, 25 and 50 mg) during 12 weeks [93]. This study enrolled patients with significant renal disease (macroalbuminuria). The authors examined the effects of ET system blockade in addition to standard care including ACEi and/or angiotensin II receptor blockers (ARBs) in patients with macroalbuminuria. In addition, the majority of the patients (73%) were under insulin therapy. The main result derived from this study was a significant decrease in 12 h urinary albumin excretion rate (UAER) with avosentan versus placebo. At the end of the study, changes in UAER ranged from −20.9% in 5 mg dosage to −29.9% in 50 mg dosage, while in placebo was of +35.5%. No correlation of blood pressure with the avosentan-induced reduction in macroalbuminuria was observed. Urinary protein excretion was also significantly reduced by avosentan treatment. However, no differences between treatment groups and placebo were observed regarding creatinine clearance, systolic blood pressure, diastolic blood pressure, body weight, and glycosylated hemoglobin. Adverse effects, mainly fluid retention, were reported in this study with a dosage-dependent manner; 32.1% of patients with fluid retention in the 50 mg dosage of avosentan versus the 3.5% in the placebo group. This was the first study to demonstrate that avosentan, in combination with standard RAS blockade treatments, reduces UAER in patients with diabetes. However, no additional antiproteinuric effect with dosages of avosentan above 25 mg was observed. Thus, one speculates that the optimal dosage in terms of risk-benefit may be equal or under 10 mg.

The ASCEND (To Determine the Effects of Avosentan on Doubling of Serum Creatinine, End Stage Renal Disease and Death in Diabetic Nephropathy) trial was a phase 3 study that was prematurely terminated because of an excess of cardiovascular events, mainly congestive heart failure and fluid overload [94]. In this study, type 2 diabetic patients with CKD stages 3–4 and overt diabetic nephropathy were randomized in three groups: avosentan 25 mg, 50 mg and placebo. Patients received treatment with ACEi and ARBs, other antihypertensive drugs, diuretics and statins. Estimated GFR declined in avosentan 50 mg group versus placebo, but no differences were found with the 25 mg group. ACR was significantly reduced in a range of 40%–50% in both avosentan groups, without differences between the two dose avosentan groups. Interestingly, when the changes in ACR were corrected for the changes in eGFR these differences persisted. However, no differences in blood pressure were observed. Importantly, mortality and adverse effects, mainly fluid overload, were significantly increased in avosentan groups. Fluid retention involves a primary effect on nephron sodium and water excretion, which, as has been described above, is mediated via ET_B_ receptors [60,62]. The authors suggest that, at higher doses, avosentan may be less effective for the ET_A_ receptor, causing sodium and water excretion as a consequence of inappropriate ET_B_ receptor blockade. In addition, the doses employed in this trial were too high, since a phase 2 study published before, demonstrated an antiproteinuric effect of avosentan at lower doses and caused only modest fluid retention [93]. Although, end-stage renal disease (ESRD) seemed to occur less frequently with avosentan, it could not be excluded that a beneficial effect on the kidney was outweighed due to increased early mortality. Taken all together, in a population with type 2 diabetes and CKD stages 3–4, avosentan is not a viable therapeutic option. Thus, studies with avosentan in this population were stopped.

#### 4.2.3. Atrasentan

Regarding atrasentan treatment and diabetic nephropathy five clinical trials have been performed and one phase 3 clinical trial is currently ongoing. The first one was a randomized, double-blind, placebo-controlled phase 2a clinical trial that enrolled type 2 diabetic subjects receiving 0.25, 0.75, 1.75 mg of atrasentan or placebo during eight weeks [95]. In these subjects, urinary ACR was significantly reduced during the course of the treatment in 0.75 and 1.75 mg dosages. In the 0.75 mg treatment group, a significant effect was seen as early as 1 week. 42% and 35% of ACR reduction in 0.75 mg and 1.75 mg groups was observed as compared to placebo group. An early and sustained reduction of systolic and diastolic blood pressure in 0.75 mg group was seen. In this trial all subjects received concomitant RAS inhibitor treatment. Thus, *post-hoc* analysis, where subjects were dichotomized by those receiving maximal doses of RAS inhibitors versus those not receiving was performed. Results demonstrated that urinary ACR was not different among these two groups, confirming that treatment effect of atrasentan is present regardless of RAS inhibition. The main adverse effect observed was dose-dependent peripheral edema: 46% of edema in 1.75 mg group, 18% in 0.75 mg group, 14% in 0.25 mg group and 9% in placebo group. Only one patient presented a serious adverse effect (accelerated hypertension and diastolic heart failure); it was seen in the 0.75 mg group. Interestingly, this subject showed a baseline N-terminal pro-brain type natriuretic peptide (NT-pro BNP) >20-fold higher than normal before receiving atrasentan. The edema was less observed than in the avosentan studies [93,94]. One may surmise that this effect might be ascribed to the low specificity of avosentan to ET_A_ receptor as compared to atrasentan. Further analysis by Andress *et al.* [96] determined that variables associated with higher risk of edema were 1.75 mg treatment group and baseline urinary ACR. In this study, markers of inflammation and renal injury were assessed. Serum measurements of CRP and IL-6 and urine measurements of MCP-1 and TGFβ found no statistically significant differences in these inflammation markers between treatments and placebo groups. The urinary NGAL markers of renal injury rose in the placebo group, while it fell with atrasentan, being of significance in the 1.75 mg group. Interestingly, no changes in ET-1 serum levels were observed, suggesting that the lack of change may be due to minimal ET_B_ receptor blockade by atrasentan.

Recently, data from two identically designed phase 2b (NCT01356849 and NCT01424319), randomized, double-bind, parallel-designed, placebo-controlled studies were pooled for analysis by de Zeeuw *et al.* [97]. In this study 0.75 mg/day, 1.25 mg/day of atrasentan or placebo was given to type 2 diabetic patients with nephropathy during 12 weeks. Significant decrease in albuminuria for 0.75 mg/day (35.5% reduction) and 1.25 mg/day group (38.6% reduction) was observed. Patients treated with 1.25 mg/day of atrasentan decreased urinary ACR and 30 days of follow-up after drop-off the treatment returned to baseline. As happened in the prior study, systolic and diastolic blood pressures fell significantly in both atrasentan groups. Only a minor correlation was found between systolic blood pressure changes and albuminuria changes in both atrasentan groups. Interestingly, atrasentan had an effect on serum lipids, lowering mean total cholesterol, LDL cholesterol and triglycerides over the 12 weeks treatment period. Minor correlation was found between albuminuria and lipid changes, but only in the 0.75 mg/day group. High dose of atrasentan significantly increased body weight compared to placebo, but prevalence of edema was comparable between baseline and after 12 weeks treatment. Although not significant, a higher number of patients discontinued the study due to fluid retention-related events after treatment with 1.25 mg/day (*n* = 8) of atrasentan compared to the 0.75 mg/day (*n* = 2) or placebo (*n* = 0) groups [97].

Currently, a phase 3 clinical trial (Study of Diabetic Nephropathy with Atrasentan, SONAR) has just started. The objective of the study is to evaluate the effect of atrasentan compared with placebo on time to doubling of serum creatinine or the onset of ESRD in subjects with type 2 diabetes and nephropathy that are treated with the maximum tolerated labelled daily dose of a RAS inhibitor. Within this trial, type 2 diabetic patients with CKD stages 3–4 and overt diabetic nephropathy are being studied. Of note, that all of them are under ACEi or ARBs and diuretic therapy. In addition, BNP levels should be less than or equal to 200 pg/mL for entry into the run-in period phase of the study. With these strategies the development of edema observed with other compounds and trials may be abrogated.

## 5. Conclusions

ET receptors are located within the kidney, in glomerulus and renal tubules as well as in renal microcirculation, becoming of great importance in regulating kidney function. Animal models have demonstrated the renoprotection of the ET receptors blockade, primarily of the selective ET_A_ receptor, in terms of decreased urinary albumin excretion, inflammatory markers and podocyte loss. In humans with DKD, it has been demonstrated that RAS blockade (both ACE inhibitors and angiotensin II receptor blockers) slows down the progression of diabetic nephropathy. However, the only partial effectiveness of these agents means that new therapeutic strategies are still needed to slow down or prevent progression to ESRD. The studies and clinical trials performed suggest that endothelin receptor antagonists, mainly ET_A_ receptor antagonists, may become a new therapeutic tool in DKD. However, fluid retention has been found as a common adverse effect in clinical trials with ET receptor antagonists. Therefore, regardless of the endothelin receptor antagonist used, careful attention must be paid to patients undergoing the study: enrollment of patients with congestive heart failure or elderly patients, who may not tolerate fluid retention, should be avoided. In addition, dosages, and diuretics used should be adjusted carefully, particularly early in the course of ET receptor antagonist treatment, which can substantially mitigate fluid retention.
